# Targeted Alpha-Particle Radiotherapy and Immune Checkpoint Inhibitors Induces Cooperative Inhibition on Tumor Growth of Malignant Melanoma

**DOI:** 10.3390/cancers13153676

**Published:** 2021-07-22

**Authors:** Mengshi Li, Dijie Liu, Dongyoul Lee, Yinwen Cheng, Nicholas J. Baumhover, Brenna M. Marks, Edwin A. Sagastume, Zuhair K. Ballas, Frances L. Johnson, Zachary S. Morris, Michael K. Schultz

**Affiliations:** 1 Viewpoint Molecular Targeting, Inc., Coralville, IA 52241, USA; mengshi-li@viewpointmt.com (M.L.); dijie-liu@viewpointmt.com (D.L.); nicholas-baumhover@viewpointmt.com (N.J.B.); brenna-marks@viewpointmt.com (B.M.M.); edwin-sagastume@viewpointmt.com (E.A.S.); frances-johnson@viewpointmt.com (F.L.J.); 2 Department of Radiology, University of Iowa, Iowa City, IA 52242, USA; dongyoul-lee@uiowa.edu; 3 Interdisciplinary Graduate Program in Human Toxicology, University of Iowa, Iowa City, IA 52242, USA; yinwen-cheng@uiowa.edu; 4 Department of Pathology, University of Iowa, Iowa City, IA 52242, USA; 5 Department of Internal Medicine, University of Iowa, Iowa City, IA 52242, USA; zuhair-ballas@uiowa.edu; 6 Department of Human Oncology, School of Medicine and Public Health, University of Wisconsin-Madison, Madison, WI 53705, USA; zmorris@humonc.wisc.edu; 7 Department of Pediatrics, University of Iowa, Iowa City, IA 52242, USA; 8 Department of Chemistry, University of Iowa, Iowa City, IA 52242, USA

**Keywords:** immunotherapy, alpha-particle radiotherapy, immunogenic cell death, immune checkpoint inhibitors, melanoma

## Abstract

**Simple Summary:**

Radiation therapy and immune checkpoint inhibitors (ICIs) have been demonstrated to cooperatively activate adaptive anti-tumor immunity with curative potential in preclinical models of melanoma. Receptor-targeted radionuclide therapy can be systemically injected to selectively deliver ionizing radiation to tumor sites throughout the body, potentially rendering all tumor sites more susceptible to anti-tumor immune response. In this study, we demonstrated the feasibility of delivering alpha-particle radiation to murine melanoma tumors using a ^212^Pb radiolabeled peptide [^212^Pb]VMT01 that targets the melanocortin 1 receptor (MC1R). Our data showed anti-tumor cooperation between [^212^Pb]VMT01 and ICIs in melanoma, mediated by induction of tumor-specific immunity. The immunogenicity of [^212^Pb]VMT01 in melanoma was also evidenced by enhanced tumor infiltrating lymphocytes and tumor vaccination assays.

**Abstract:**

Radiotherapy can facilitate the immune recognition of immunologically “cold” tumors and enhance the efficacy of anti-PD-1 and anti-CTLA-4 immune checkpoint inhibitors (ICIs) in melanoma. Systemic administration of receptor-targeted radionuclide therapy has the potential to selectively deliver radionuclides to multiple tumors throughout the body in metastatic settings. By triggering immunologic cell death and increasing the immune susceptibility of surviving tumor cells in these locations, targeted radionuclide therapies may overcome resistance to ICIs and render immunologically “cold” tumors throughout the body responsive to ICIs and immunologically “hot”. Here, we show the anti-tumor cooperation of targeted α-particle radionuclide therapy (α-TRT) and ICIs in preclinical models of melanoma. Melanocortin 1 receptor (MC1R)-targeted radiopeptide [^212^Pb]VMT01 was employed to deliver α-radiation to melanoma tumors in mice. A single injection of 4.1 MBq [^212^Pb]VMT01 significantly slowed the tumor growth of B16-F10 melanoma and the combination of [^212^Pb]VMT01 and ICIs induced a cooperative anti-tumor effect leading to 43% complete tumor response with no sign of malignancy on autopsy. Animals with complete response developed anti-tumor immunity to reject further tumor inoculations. This therapeutic cooperation was completely abolished in RAG1 KO mice, which are deficient in T-cell maturation. In addition, the anti-tumor cooperation was compromised when fractionated [^212^Pb]VMT01 was used in the combination. We also demonstrated that [^212^Pb]VMT01 induced immunogenic cell death in tumor vaccination assays and in vitro exposure to [^212^Pb]VMT01 sensitized immunotolerant melanoma to ICIs treatment in vivo. Enhanced tumor infiltrating CD3^+^, CD4^+^, CD8^+^ lymphocytes were observed following injection of 1.4 MBq [^212^Pb]VMT01. Overall, we demonstrated anti-tumor cooperation between α-TRT and ICIs in melanoma that is mediated by tumor specific immunity.

## 1. Introduction

Melanoma is a potentially aggressive form of skin cancer, with an estimated 100,350 new cases and 6850 deaths in the US in 2020 [1]. The identification of signature genetic mutations and immune escape mechanisms have led to breakthrough mitogen-activated protein kinase (MAPK) inhibitors (i.e., BRAF inhibitors, MEK inhibitors) [2] and immune checkpoint inhibitors (ICIs; anti-PD-1, anti-CTLA-4, anti-PD-L1) [3,4,5,6]. Inhibition of CLTA-4 and PD-1 facilitates T-cell activation via different mechanisms. The immunosuppressive CLTA-4 machinery primarily competes against CD28 for B7 molecules on antigen-presenting cells [7,8], and the blockade of CTLA-4 facilitates T-cell priming at secondary lymphoid tissues (e.g., spleen, lymph nodes) and depletes intratumoral regulatory T cells (T_reg_) [9]. On the other hand, the inhibition of PD-1/PD-L1 signaling reverses T-cell exhaustion at the sites of effector T-cell function (e.g., CD8^+^ cytotoxic T cells and CD^4+^ type 1 helper T cells) in nonlymphoid tissues [10,11,12]. Clinical studies have reported robust efficacy of the combination of nivolumab (anti-PD1) and ipilimumab (anti-CTLA-4) in patients with metastatic melanoma. A complete response rate of 22% for the combination has been reported, compared to 19% for nivolumab, and 6% ipilimumab alone [13]. Despite the demonstrated efficacy of ICIs in treating metastatic melanoma, approximately half of all patients will not respond to this treatment, and a small minority of patients will derive durable survival benefit (five-year survival < 36%) [13].

Emerging evidence suggests that further improvements therapy outcomes can be achieved by combining ICIs with other anti-cancer therapeutics in melanoma (e.g., chemotherapy, radiotherapy and targeted therapies) [14,15]. Among these approaches, ionizing radiation has been well characterized as a potent inducer for immunogenic cell death that leads to enhanced tumor antigen cross-presentation by dendritic cells and activation of tumor-specific cytotoxic T cells [16,17,18,19,20]. These responses can be augmented by combining radiotherapy with systemic immunotherapy agents, such as immune checkpoint inhibitors [21,22,23,24,25]. The immunosensitizing effects of ionizing radiation have long been investigated, particularly within the context of induction of an “abscopal” effect. The abscopal effect of radiotherapy, i.e., a phenomenon in which the irradiation of primary tumor induces regression of distal untreated tumors, was first described in the 1950s [26] and numerous studies have explored the combination of immunotherapies and radiotherapy using single-site irradiation strategy since that time [27,28]. However, apparent manifestations of abscopal effects with external beam radiation alone have been documented in only 46 individual cases identified in 31 studies over 50 years [29]. Evidence suggests that a major underlying reason for this is that intertumoral heterogeneity of antigen expression (e.g., between primary and distal malignancy), together with the immunosuppressive nature of the non-radiated tumor microenvironment(s), causes suboptimal efficacy of the single-site irradiation approach in activating an effective systemic anti-tumor immune response [30,31,32,33,34,35]. On the other hand, the delivery of radiation to multiple tumor sites, or ideally to all tumor sites, has the potential to reach all immunologically “hot” tumor sites and enhance the recognition of diverse tumor-associated antigens that may better enable adaptive immune-mediated destruction of heterogeneous of metastatic melanoma tumors [36,37,38,39,40,41].

One promising approach to systemically deliver radiation to multiple tumor sites is targeted radionuclide therapy (TRT), whereby a tumor-targeting ligand (e.g., small molecule, peptide, antibody) is labeled with a radionuclide. The radiolabeled ligand specifically binds with a tumor cell surface antigen that is overexpressed in tumor cells relative to normal cells [42,43,44]. Upon binding to tumor cells, the radiolabeled ligand delivers cell-killing beta (β)- or alpha (α)-particles to tumor, while sparing normal tissues. Melanocortin 1 receptor (MC1R) has long been investigated as a promising target for metastatic melanoma drug delivery due to its overexpression in melanoma cells and relatively low expression in normal cells [45,46,47,48]. One particularly attractive avenue for this drug-targeting paradigm has been the use of radiolabeled synthetic peptide analogs of α-melanocyte stimulating hormone (α-MSH) to deliver radionuclides to the melanoma tumor microenvironment via binding with MC1R. Our group has previously reported several “click” cyclized synthetic α-MSH analogs that bind to MC1R with nanomolar affinity [49]. In this study, we demonstrate that the combination of ICIs and MC1R-targeted α-particle TRT (α-TRT), using “click” cyclized α-MSH variant VMT01 radiolabeled with lead (Pb) isotope ^212^Pb to deliver α-particles, induce a cooperative anti-tumor effect in immunocompetent C57BL6 mice bearing syngeneic murine melanoma tumors (B16-F10), achieving a complete response rate of 43%. The anti-tumor cooperation of the combination is shown to be mediated by tumor specific immunity that is activated by [^212^Pb]VMT01.

## 2. Materials and Methods

### 2.1. Cell Lines, Reagents, Materials, and Animals

B16-F10, B16-F0, YUMM1.7 cells were obtained from ATCC and used within passage 10. All cells were culture in complete growth media including DMEM medium with 10% FBS, 100 units/mL Pen Strep, and 100 units/mL streptomycin. All cells were grown at 37 °C in a humidified atmosphere (5% CO_2_). Radiometals ^203^Pb chloride was obtained from Lantheus Medical Imaging (North Billerica, MA, USA). The ^224^Ra/^212^Pb generator was provided by Oak Ridge National Laboratory (Oak Ridge, TN, USA). Pb-specific resin was obtained from Eichrom Technologies (Lisle, IL, USA). Anti-mouse CTLA-4 (Clone 9H10), anti-mouse PD-1 (Clone 29F.1A12), and rat IgG2a isotype control were purchased from BioXCell (Lebanon, NH, USA). Fluorophore-conjugated antibodies used in FACS were purchased from Biolegend (San Diego, CA, USA). Matrigel was purchased from Corning (Corning, NY, USA). StrataX C-18 SPE cartridges were obtained from Phenomenex (Torrance, CA, USA). All other chemicals were purchased from Thermo Fisher Scientific (Waltham, MA, USA). C57BL6 mice and Rag1 KO mice were obtained from The Jackson Laboratory (Bar Harbor, ME, USA). Athymic nude mice were purchased from Envigo (Indianapolis, IN, USA). All animal studies were performed in accordance with the Guide for the Care and Use of Laboratory Animals.

### 2.2. Radiolabeling, In Vivo Biodistribution and Kidney Dosimetry 

To determine the injected radioactivity of [^212^Pb]VMT01, [^203^Pb]VMT01 was used as a surrogate in to determine the biodistribution of Pb-labeled VMT01. Radiolabeling of VMT01 was carried out according to published methods [50]. Briefly, ^203^Pb^2+^ was purified on 50 mg Pb-resin (Eichrom Technologies, Lisle, IL, USA) and eluted into reaction vessel using 0.5 M sodium acetate (NaOAc) pH = 6 buffer. The reaction vessel contained 20 nmole VMT01 peptide precursor and 0.29 mL of 0.5 M sodium acetate (NaOAc) pH = 4 buffer to adjust final pH to 5.4. The reaction solution was heated under 85 °C for 30 min. After reactions, free ^203^Pb was removed by StrataX C-18 SPE cartridge (Phenomenex, Torrance, CA, USA) and final products were collected in 50% EtOH in saline. Biodistribution of [^203^Pb]VMT01 was determined in a B16-F10 murine melanoma xenograft model in athymic nude mice. B16-F10 xenograft was developed by the subcutaneous (SC) injection of 2 × 10^5^ B16-F10 cells at the left shoulder in 100 µL 50% Matrigel in complete growth media. Then, 74 KBq of [^203^Pb]VMT01 (5.4 pmole) were administered via tail vein injection (2 male and 2 female at each time point) in 100 µL of saline with less than 10% EtOH content. At 0.5, 1.5, 3, 6, and 24 h post-injection, animals were euthanized, and organs of interest were harvested and weighed. Radioactivity in tumor and organs was measured on a Cobra II automated gamma counter. To determine the injected radioactivity of [^212^Pb]VMT01, time-integrated accumulation of radioactivity in kidney was calculated by the trapezoidal method up to 48 h accounting for approximately 5 half-lives of ^212^Pb (t_1/2_ = 10 h). The value for 48 h was extrapolated from the last 3 time points (3, 6, and 24 h) of the biodistribution by one phase exponential decay with least squares fitting method (GraphyPrism V7). The average kidney volume was assumed to be 0.33 cm^3^ for C57BL6 mice. The DigiMouse voxel phantom model (28 g; normal male mouse) was used to calculate s-value and absorbed dose in kidney from the decay of ^212^Pb using the Particle and Heavy Ion Transport code System (PHITS) software version 2.76 (Japan Atomic Energy Agency, Tokai, Japan) as we previously reported [51]. The voxel size of the model was adjusted to have the same kidney volume as the averaged value (0.33 cm^3^). The elemental composition of the kidney and the mass density was assumed to be identical as the human adults’ values obtained from the International Commission on Radiation Units and measurements (ICRU) report 46 [52]. The injected radioactivity of [^212^Pb]VMT01 was determined using 11 Gy dose deposition in the kidney as threshold in this study as guided by a previous safety study of [^213^Bi]DOTATATE [53].

### 2.3. Combination Therapy of Immune Checkpoint Inhibitors and [^212^Pb]VMT01

Cooperative anti-tumor efficacy between ICIs and [^212^Pb]VMT01 was determined in C57BL6 mice bearing B16-F10 melanoma. Preparation of [^212^Pb]VMT01 was described in our previous publication [54]. In general, ^212^Pb^2+^ was eluted from ^224^Ra/^212^Pb generator (US Department of Energy, Oak Ridge, TN, USA) with 2 M HCl. The ^212^PbCl_2_ eluate was purified on Pb-resin and reacted with 20 nmole VMT01 as described above. After reactions, free ^212^Pb^2+^ was removed by C-18 SPE cartridge and a final dose was collected in 50% EtOH in saline. In C57BL6 mice bearing a B16-F10 tumor, therapies were initiated when the tumor size reached 50 mm^3^ (4–5 days post-inoculation). For [^212^Pb]VMT01 monotherapy, 4.1 MBq [^212^Pb]VMT01 (0.3 nmole) was administered via the tail vein in 100 µL of saline containing 8 mg of DL-lysine to further reduce the accumulated radiation dose in kidney. ICIs including 200 µg of anti-mouse CLTA4 and 200 µg anti-mouse PD-1 were administered twice a week via IP injection. The combination of ICIs and [^212^Pb]VMT01 was administered concurrently on day 0, followed by routine doses of ICIs given twice a week via IP injection. Control animals were treated with 200 µg rat IgG2a isotype control via IP injection. Upon conclusion of the study, tumor re-challenge was conducted in animals that demonstrated complete tumor regression as results from combination of [^212^Pb]VMT01 + ICIs. These animals were removed from study on 80 days and kept in animal housing facility for 7 days, followed by tumor re-challenge using SC injection of 50,000 naïve B16-F10 cells on Day 87. Animals were monitored for extra 60 days post-inoculation.

To determine the impact of dosing regimen of [^212^Pb]VMT01 on the effectiveness of [^212^Pb]VMT01 as monotherapy as well as in combination with ICIs, total 4 MBq [^212^Pb]VMT01 was delivered via tail vein injection over three fractions within 6 days (*n* = 7), including 2 MBq on day 0, 1 MBq on day 3, and 1 MBq on day 6. Each fraction of [^212^Pb]VMT01 was administered in 100 µL of saline containing 8 mg of DL-lysine. Combination of [^212^Pb]VMT01 and ICIs started concurrently on day 0, by IP injection of 200 µg anti-mouse CLTA4 and 200 µg anti-mouse PD-1, along with the first 2 MBq fraction of [^212^Pb]VMT01. Control and ICIs monotherapy cohorts were treated with IP injection of IgG isotype control and anti-mouse CLTA4/anti-mouse PD-1, respectively, as described above. Tumor growth was monitored by measuring tumor size twice a week by length (L) and width (W) using the following equation:

Volume = L × W^2^/2

Animals were removed from the study when tumor size reached 1500 mm^3^; tumor ulcerations appeared; body weight loss was more than 20% compared with initial weight; or other significant toxicity was observed. To evaluate the effectiveness of treatments in each cohort, median overall survival (MOS) and tumor-doubling time were compared with initial tumor size on day 0. 

### 2.4. Combination of Immune Checkpoint Inhibitors and Single Dose of [^212^Pb]VMT01 in Rag1 KO Mice

To investigate if the immunogenicity of [^212^Pb]VMT01 was mediated by adaptive immune response, the combination of [^212^Pb]VMT01 and ICIs was applied to B6.129S7-Rag1^tm1Mom^/J mice (i.e., Rag1 KO mice) mice. Due to the genetic modification, Rag1 KO mice do not produce mature B and T lymphocytes therefore are considered “non-leaky” immune deficiency. B16-F10 melanoma xenograft was developed in Rag1 KO mice by SC injection of 2 × 10^5^ of B16-F10 cells on left shoulder. Therapies were initiated when tumor size neared 50 mm^3^. Monotherapy of [^212^Pb]VMT01 was delivered as single injection of 4.1 MBq [^212^Pb]VMT01 via tail vein. ICIs (i.e., 200 µg anti-CTLA-4 and 200 µg anti-PD-1) were administered via IP injection twice a week. Combination of ICIs and [^212^Pb]VMT01 was administered concurrently on day 0. Control cohorts were treated with IP injection of IgG isotype control antibody twice a week. Following the treatments, tumor size was measured twice a week by length (L) and width (W) as described above.

### 2.5. Vaccination and Tumor Re-Challenge

To determine the activation of anti-tumor immune response by [^212^Pb]VMT01, [^212^Pb]VMT01 treated melanoma cells were injected in C57BL6 mice as cell-based vaccine to stimulate anti-tumor immunity. B16-F10 and B16-F0 cells were kept under 37 °C and 5% CO_2_ to grow until 50–80% confluency in 60 mm petri dishes. The 0.6 MBq [^212^Pb]VMT01 was added to 5 mL total growth media and incubated for 24 h before removal of radioactive media. After treatment, cells were cultured in fresh media for another 24 h before further inoculation in C57BL6 mice. Upon vaccination in C57BL6 mice, 2 × 10^6^ [^212^Pb]VMT01 treated B16-F10 or B16-F0 cells were subject to SC inoculation in 100 µL 50% Matrigel in total culture media at the left shoulder (*n* = 7–8). In control animals, in 100 µL 50% Matrigel in total culture media without any cells, SC was injected at the left shoulder. Then, 7 days post-vaccination, mice were re-challenged with SC inoculation of 50,000 naïve B16-F10 or B16-F0 at the contralateral right shoulder. Tumor progression was monitored by measuring length (L) and width (W) twice a week.

### 2.6. Generation of Immunosensitized Syngeneic Melanoma Cells by [^212^Pb]VMT01 

The immunogenicity of [^212^Pb]VMT01 was determined in immunotolerant syngeneic mouse melanoma cells lines B16-F10 and YUMM1.7. Immunosensitized melanoma cells were generated from B16-F10 and YUMM-1.7 cells using modified methods based on previous publication [55]. YUMM-PR (post radiation) and B16-PR cells were generated by treating naïve YUMM-1.7 and B16-F10 cells with 0.22 MBq [^212^Pb]VMT01 for 24 h in complete growth media (DMEM medium with 10% FBS, 100 units/mL Pen Strep, and 100 units/mL streptomycin) in 35 mm petri dishes. After [^212^Pb]VMT01 treatment, YUMM-PR and B16-PR cells were cultured under 37 °C and 5% CO_2_ in complete culture media for extra 2 weeks, allowing for full recovery of irradiated cells. Culture media were replaced every three days to remove floating cells. After two weeks, xenografts of YUMM-PR and B16-PR tumor were developed by SC inoculation of 1 × 10^6^ YUMM-PR and 1 × 10^5^ B16-PR cells in female C57BL6 mice (*n* = 5) as described above. ICIs treatment (i.e., 200 µg anti-mouse CLTA-4 and 200 µg anti-mouse PD-1) was initiated when YUMM-PR and B16-PR tumors reached 100 mm^3^ and 50 mm^3^, respectively. ICIs and rat IgG isotype control were administered via IP injection twice a week. 

### 2.7. FACS Analysis of Tumor Infiltrating Lymphocytes

[^212^Pb]VMT01-induced tumor infiltrating lymphocytes (TIL) in B16-F10 was analyzed by FACS. In C57BL6 mice bearing B16-F10 melanoma, 1.4 MBq of [^212^Pb]VMT01 was injected via tail vein (*n* = 4) when tumor size reached 100 mm^3^. Control animals were treated with isotonic saline (*n* = 5). Then, 7 days after treatments, animals were euthanized, and tumors were exercised for FACS analysis. Briefly, tumor samples were placed in GentleMACS™ C-tubes (Miltenyibiotec) containing 3 mL of ice-cold RMPI media. Tumor samples were homogenized on gentleMACS™ Dissociator (Miltenyibiotec) and filtered through 70-micron cell strainer to get single cell suspension. Then, 15 mg of homogenized samples was transferred to 12 × 75 mm tubes and washed twice with ice-cold PBS. Cells were stained for live/dead using Zombie Aqua dye diluted at 1:100 in 100 µL PBS and incubated at room temperature for 15 min. To stain surface markers, cells were first washed in FACS buffer (PBS, 2% BSA, 1 mM EDTA, 0.1% sodium azide) twice and then staining in 100 µL of FACS buffer containing 0.5–1 µg of anti-mouse CD45-PerCP-Cy5 (103132, Biolegend, San Diego, CA, USA), anti-mouse CD3-APC (100235, Biolegend), anti-mouse CD19-PE-Cy7 (115519, Biolegend), anti-mouse CD4-APC-Cy7 (100413, Biolegend), and anti-mouse CD8-FITC (100706, Biolegend). Cells were incubated under room temperature for 15 min before washed with FACS buffer twice. Finally, stained cells were fixed in 200 uL 0.5% formaldehyde and analyzed on a BD Becton Dickinson LSR II (VA Satellite Lab) flow cytometer at the Flow Cytometry Facility at the University of Iowa. 

## 3. Results

### 3.1. Radiolabeled Peptide VMT01 Delivers Ionizing Radiation to Melanoma Cells via Specific Binding to MC1R

**Figure 1:**
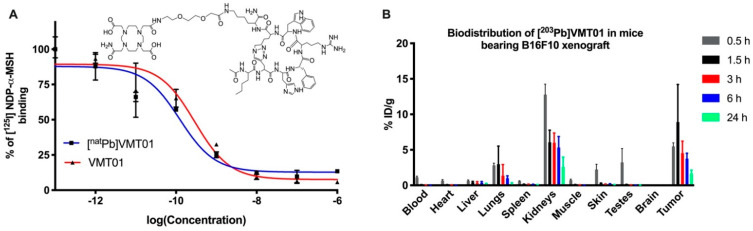
MC1R-targeted peptide ligand direct ionizing radiation to melanoma via binding with the receptor. (**A**) Competitive binding of VMT01 and [^nat^Pb]VMT01 against [^125^I]NDP-α-MSH in B16-F10 cells; (**B**) Biodistribution of [^203^Pb]VMT01 in athymic nu/nu mice bearing B16-F10 melanoma (*n* = 2 male and *n* = 2 female at each time point); Data were expressed as percent of injected dose per gram of tissue (%ID/g) ± SD.

Radiolabeled synthetic α-MSH analog VMT01 (Figure 1A) was employed to deliver Pb isotopes ^203^Pb and ^212^Pb to melanoma cells via binding with MC1R. Competitive binding assays against [^125^I]NDP-α-MSH were conducted in B16-F10 to determine the binding affinity of VMT01 and [^nat^Pb]VMT01. Further, 0.29 and 0.15 nM IC_50_ were identified for VMT01 and [^nat^Pb]VMT01, respectively (Figure 1A). In vivo biodistribution of Pb-labeled VMT01 was determined using [^203^Pb]VMT01 in female athymic nude mice bearing MC1R-positive B16-F10 melanoma. Rapid accumulation of [^203^Pb]VMT01 in B16-F10 melanoma was observed (Figure 1B). Accumulation of [^203^Pb]VMT01 in B16-F10 tumors was 5.5, 8.9, 4.5, 3.8, and 1.7 percent injection dose per gram (%ID/g) at 0.5, 1.5, 3, 6, and 24 h post administration, respectively (Table 1). Excessive [^203^Pb]VMT01 was cleared from circulation rapidly, with 1.1%ID/g residual radioactivity in blood at 0.5 h post-injection (Figure 1B, Table 1). Off-target accumulation was primarily localized in kidney, with 12.8, 6.1, 6.0, 5.3, and 2.6%ID/g at 1.5, 3, 6, and 24 h post injection (Figure 1B, Table 1). Cumulative radioactive decays of [^212^Pb]VMT01 in kidney was integrated using the biodistribution data of [^203^Pb]VMT01 and corrected with decay half-life of ^212^Pb (t_1/2_ = 10.64 h). The calculated s-value for [^212^Pb]VMT01 in kidney was 2.84E-06 Gy/Bq-s in kidney. To maintain the dose deposition in kidney below 11 Gy for therapeutic applications, the upper limits of injected radioactivity for [^212^Pb]VMT01 were estimated to be 4.1 MBq.

### 3.2. Combination of ICIs and [^212^Pb]VMT01 Induces Significant Tumor Inhibition and Lasting Anti-Tumor Immunity

**Figure 2:**
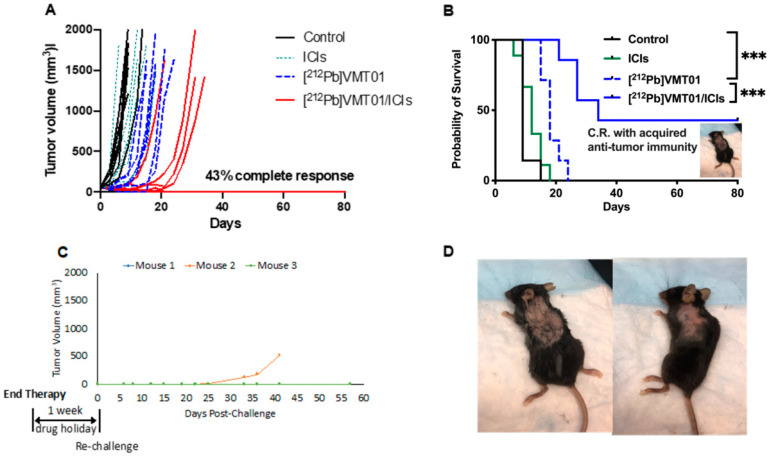
Anti-tumor effect from combination of single dose [^212^Pb]VMT01 α-TRT and ICIs B16-F10 melanoma. (**A**) Individual tumor volume in animals after treatments of rat IgG isotype control (control), single dose of 4.1 MBq [^212^Pb]VMT01; 200 µg of anti CTLA-4 + 200 µg anti PD-1 (ICIs), and combination of [^212^Pb]VMT01 and ICIs (*n* = 7 in each group); (**B**) Overall fractional survival in C57BL6 mice bearing B16-F10 melanoma that received rat IgG isotype control, [^212^Pb]VMT01, ICIs, and combination of [^212^Pb]VMT01 and ICIs (*n* = 7 in each group); Statistic analysis was performed using Gehan-Breslow-Wilcoxon test: *** *p* < 0.001; (**C**) Individual tumor growth of B16-F10 tumor re-challenge in mice with complete response to combination of [^212^Pb]VMT01 and ICIs; (**D**) Mice rejected further implantation of B16-F10 melanoma tumor up to 60 days after re-challenge.

To determine the potential cooperative anti-tumor effects that could be induced by combining MC1R-targeted α-TRT and ICIs, [^212^Pb]VMT01 was administered as a monotherapy or in combination with dual ICIs (i.e., anti-CLTA-4 + anti-PD-1) in immunocompetent C57BL6 mice bearing B16-F10 syngeneic murine melanoma tumors (*n* = 7 in each cohort). Tumors were induced by a subcutaneous inoculation of 2 × 10^5^ B16-F10 cells on the left shoulder. Therapies were initiated when tumors reached 60 ± 13 mm^3^. In the control cohort, tumor-volume endpoint (1500 mm^3^) was reached shortly after the initiation of the experiment (<10 days). Further, 86% of animals that received IgG isotype control were removed from study within 10 days due to uncontrolled tumor growth (Figure 2A). The median overall survival (MOS) of control animals was nine days (Figure 2B). Dual ICIs injected twice a week did not provide significant control on tumor growth, consistent with these tumors being “immunologically cold”. The MOS (12 days) in the ICIs alone treatment group was not significantly different from the control group (Figure 2B). Median tumor-doubling time was not identified in these two groups due to rapid uncontrolled tumor growth. On the other hand, a single injection of 4.1 MBq [^212^Pb]VMT01 significantly suppressed the growth of B16-F10 tumor in all treated animals. In these animals, it took median 10 days to reach doubled tumor size compared with tumor size day 0 (Figure 2A). The MOS was also extended to 18 days (Figure 2B, *p* < 0.0001 vs. control). More significant inhibition of tumor growth was observed in mice treated with a combination of [^212^Pb]VMT01 and ICIs. In this cohort of animals, the treated tumors took 24 days to reach doubled size from day 0 (Figure 2A). MOS was also prolonged to 34 days (Figure 2B, *p* < 0.001 vs. [^212^Pb]VMT01 monotherapy). Importantly, 100% (seven in seven) animals responded to this combination therapy, with 43% (three in seven) showing complete tumor regression and the surviving mice remained tumor-free until the conclusion of the experiment on day 80. No weight loss or other significant toxicity was observed in these animals (Supplemental Figure S1). 

After the conclusion of therapy, adaptive anti-tumor immunity was determined in animals that had achieved complete responses. These mice were re-challenged by SC inoculation of 50,000 naïve B16-F10 cells after conclusion of the therapy study (one week ICIs drug holiday). Remarkably, while control B16-F10 tumors are generally aggressive and grow rapidly, inoculations of the naïve B16-F10 cells in these animals were either significantly attenuated or did not grow within the study period. Of the three mice in this cohort, two animals completely rejected tumor inoculation and maintained tumor-free status for and additional 60 days. Further, tumor development was significantly attenuated in the third mouse in this cohort, with the tumor slowly developing and emerging approximately 30 days after implantation (Figure 2C,D). These data suggest that [^212^Pb]VMT01 and ICIs combine to induce a cooperative tumor-inhibition effect that can lead to complete tumor regression, where monotherapies of ICIs and [^212^Pb]VMT01 fall short. In addition, the anti-tumor immunity acquired during the course of the combination therapy immunizes the mice to reject further tumor implantation or to significantly inhibit tumor growth.

### 3.3. Combination of ICIs with Fractionated [^212^Pb]VMT01 Compromised the Cooperative Anti-Tumor Effects Observed for the Single-Dose α-TRT Plus ICIs Combination

**Figure 3:**
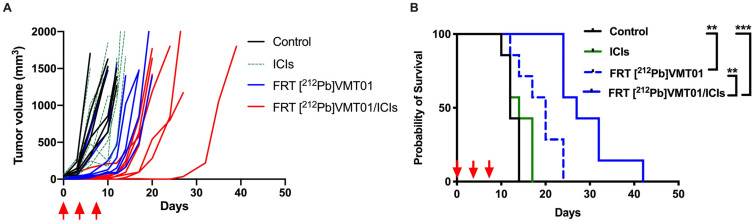
Fractionated dose of [^212^Pb]VMT01 α-TRT in combination with ICIs in C57BL6 mice bearing B16-F10 melanoma. (**A**) Individual tumor volume in each group of animals after treatments were initiated. Treatments included rat IgG isotype control (control), fractionated 4 MBq [^212^Pb]VMT01 (FRT [^212^Pb]VMT01); 200 µg of anti CTLA-4 + 200 µg anti PD-1 (ICIs), and combination of FRT [^212^Pb]VMT01 and ICIs (*n* = 7 in each group); (**B**) Overall fractional survival in B16-F10 tumor xenograft models that received control IgG, ICIs, FRT [^212^Pb]VMT01 and combination of FRT [^212^Pb]VMT01 and ICIs; Statistic analysis was performed using Gehan-Breslow-Wilcoxon test: ** *p* < 0.01; *** *p* < 0.001.

To refine our understanding of the combination of [^212^Pb]VMT01 and ICIs, the impact of dosing regimen of [^212^Pb]VMT01 as monotherapy and in combination with ICIs was examined. For this assessment, [^212^Pb]VMT01 was administered using a dosing regimen of a total 4.0 MBq over three fractions (2 + 1 + 1 MBq) injected in C57BL6 mice via tail vein (*n* = 7), with an interval of three days between each administration. The [^212^Pb]VMT01 was administered as monotherapy, as well as in combination with ICIs. Despite that [^212^Pb]VMT01 was administered over three fractions, fractionated [^212^Pb]VMT01 monotherapy resulted in robust inhibition of B16-F10 tumor growth. Compared with the rapid tumor growth in control and ICIs cohorts, it took 12 days to reach doubled tumor size in mice treated monotherapy of fractionated [^212^Pb]VMT01 (Figure 3A). The MOS in animals administered with fractionated [^212^Pb]VMT01 was improved to 20 days (*p* < 0.05 vs. control, Figure 3B). When the fractionated [^212^Pb]VMT01 regimen was applied in combination with ICIs, a clear cooperation between [^212^Pb]VMT01 and ICIs was observed. In these animal cohorts, the median tumor-doubling time was extended to 17 days, and MOS was also extended to 27 days (Figure 3A,B). Compared with monotherapies of fractionated [^212^Pb]VMT01 or ICIs, the improvement from combination of fractionated [^212^Pb]VMT01 and ICIs was significant (*p* < 0.01 vs. [^212^Pb]VMT01; *p* < 0.001 vs. ICIs). However, all animals treated with combination therapy eventually developed progressive tumors and no complete tumor regression was observed (Figure 3B). These results indicate that both single and fractionated injection of [^212^Pb]VMT01 monotherapy efficiently attenuated MC1R-postive melanoma tumor, but only single injection of [^212^Pb]VMT01 induced an immune response that led to complete tumor regression in combination with ICIs.

### 3.4. [^212^Pb]VMT01 Induces Anti-Tumor Immunity That Relies on the Involvement of Adaptive Immunity

**Figure 4:**
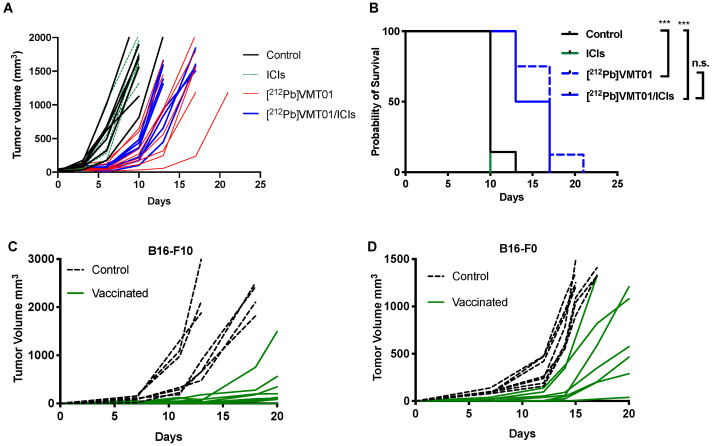
[^212^Pb]VMT01 α-TRT induces anti-tumor immune responses that rely on adaptive immunity. (**A**) individual tumor volume in RAG1 K/O mice after treatments of rat IgG isotype control (control), single dose of 4.1 MBq [^212^Pb]VMT01; 200 µg of anti CTLA-4 + 200 µg anti PD-1 (ICIs), and combination of [^212^Pb]VMT01 and ICIs (*n* = 7 in each group); (**B**) Overall fractional survival in in RAG1 K/O mice bearing B16-F10 melanoma that received IgG control, [^212^Pb]VMT01, ICIs, and combination of [^212^Pb]VMT01 and ICIs (*n* = 7 in each group); Statistic analysis was performed using Gehan-Breslow-Wilcoxon test: n.s. non-significant; *** *p* < 0.001; Cell-based vaccine using [^212^Pb]VMT01 treated B16F0 or B16-F10 cells was injected in C57BL6 mice (*n* = 7). Individual tumor growth of re-challenging (**C**) B16-F10 and (**D**) B16-F0 tumor on the contralateral side of the primary tumor.

To begin testing whether T cell maturation was necessary for the cooperative anti-tumor effect of the combination of [^212^Pb]VMT01 and ICIs, this treatment combination was administered to B6.129S7-Rag1^tm1Mom^/J mice (i.e., Rag1 KO mice) bearing B16-F10 tumors (*n* = 7), where 4.1 MBq [^212^Pb]VMT01 was administered as a single injection on day 0, as this regimen showed most significant anti-tumor effectiveness. Not surprisingly, animals in control and ICIs monotherapy cohorts rapidly reached endpoint (1500 mm^3^) due to aggressive tumor progression. Within 10 days, 100% animals in ICIs cohorts and 86% animals in control cohorts were removed (Figure 4A). On the other hand, despite the depleted adaptive immunity in Rag 1 KO mice, monotherapy of 4.1 MBq [^212^Pb]VMT01 still led to significant inhibition of growth of B16-F10 tumors (Figure 4A) and the MOS in these animals was improved to 17 days (Figure 4B). However, with the deficient adaptive immunity in the Rag1 KO mice, the benefit from combination of [^212^Pb]VMT01 and ICIs was completed abrogated. Compared with [^212^Pb]VMT01 monotherapy, combination therapy did not provide a significant improvement in therapeutic outcome (MOS = 15 days, *p* > 0.05 vs. [^212^Pb]VMT01, Figure 4A,B). These data indicate that the immunogenicity of [^212^Pb]VMT01 and anti-tumor cooperation with ICIs require intact adaptive T cell immunity. To elucidate the activation of tumor-specific immune response by [^212^Pb]VMT01, in vivo vaccination and tumor re-challenge assays were performed. Female C57BL6 mice (*n* = 7) were vaccinated by SC inoculation of 2 × 10^6^ B16-F10 or B16-F0 cells that were pre-treated with 0.6 MBq [^212^Pb]VMT01 in vitro. These [^212^Pb]VMT01-treated melanoma cells were employed as a cell-based vaccine in C57BL6 mice. Control animals were injected with 100 µL PBS subcutaneously. [^212^Pb]VMT01 treatment efficiently killed melanoma cells, as implantation of 2 × 10^6^ [^212^Pb]VMT01-treated B16-F10 and B16-F0 cells did not give rise to any tumor growth (Figure 4C,D). One week post vaccination, both immunized mice and control mice were re-challenged by SC inoculation of 50,000 naïve B16-F10 or B16-F0 cells on the contralateral side of animals. Compared with the control mice, slower progression of both B16-F10 (Figure 4C) and B16-F0 tumors (Figure 4D) was observed in vaccinated mice, indicating that [^212^Pb]VMT01 activates tumor-specific immunogenicity that produces immune protection against further tumor inoculation. 

### 3.5. [^212^Pb]VMT01 Sensitizes Immunotolerant Melanoma Cells to ICIs and Induces Tumor-Infiltrating Lymphocytes

**Figure 5:**
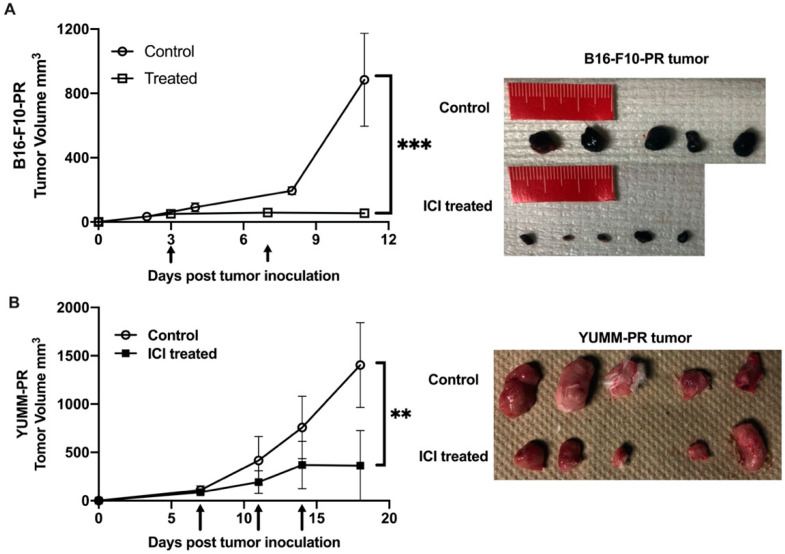
[^212^Pb]VMT01 sensitizes immunotolerant melanoma cells to ICIs treatment. [^212^Pb]VMT01-treated (**A**) B16-PR tumor and (**B**) YUMM-PR melanoma responded to ICIs treatment in C57BL6 mice. Arrows indicated ICIs treatment. ** *p* < 0.01, *** *p* < 0.001.

To determine if [^212^Pb]VMT01 changes the immunophenotype of melanoma cells, sensitization to ICIs by [^212^Pb]VMT01 was conducted in immunotolerant B16-F10 and YUMM1.7 syngeneic melanoma cells. Due to their immunotolerant nature, B16-F10 tumor did not respond to ICIs treatment as we demonstrated above. Similarly, the immunotolerance of YUMM1.7 tumor has been previously characterized [55], whereas UV radiation treatment induced accumulation of somatic mutations that sensitized YUMM1.7 tumor to ICIs treatment [55]. In this study, B16-PR (post radiation) and YUMM-PR cells were generated by treating these cells with [^212^Pb]VMT01 in vitro. After SC implantation, fast tumor growth was observed in both B16-PR and YUMM-PR tumors in female C57BL6 mice (*n* = 5). For B16-PR tumor, in mice administered with rat IgG isotype control antibody, tumor size reached 884 ± 324 mm^3^ within 11 days post inoculation (Figure 5A). For comparison, this growth rate was almost identical to naïve B16-F10 tumors in C57BL6 mice (Figure 4C), indicating the B16-PR cells had recovered from [^212^Pb]VMT01 treatment upon SC inoculation and were capable to give rise to fast-growing tumors. To determine if the B16-PR tumors are responsive to ICI treatments, mice were administered an identical ICIs therapy regimen as described for previous experiments. In this case, IP injection of ICIs significantly compromised the tumor growth of B16-PR, with average tumor size 56 ± 20 mm^3^ on day 11 (Figure 5A, *p* < 0.001 vs. control B16-PR). Similarly, with the injection of rat IgG control, YUMM-PR tumor grew to 1404 ± 438 mm^3^ within 18 days post inoculation (Figure 5B), whereas ICIs treatment significantly suppressed tumor growth of YUMM-PR tumor (Figure 5B, 361 ± 364 mm^3^, *p* < 0.01 vs. YUMM-PR control). These data indicate that [^212^Pb]VMT01 treatment sensitized these immunotolerant syngeneic melanoma cells to ICIs treatment. 

**Figure 6:**
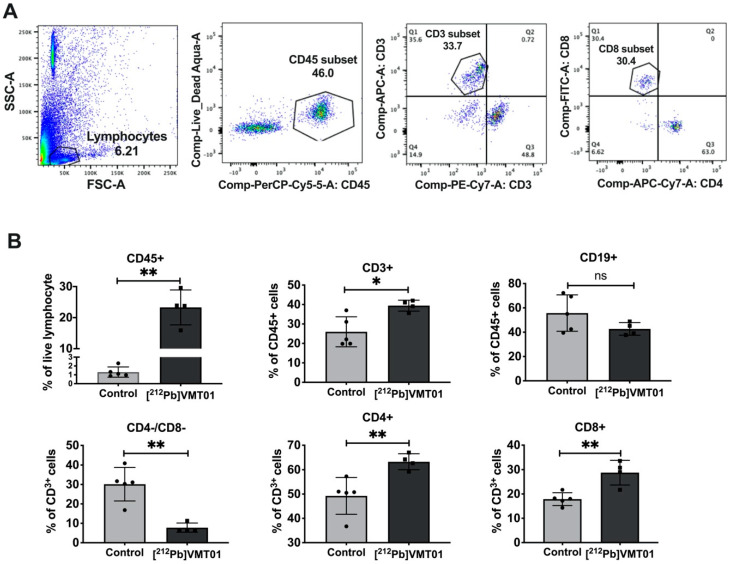
[^212^Pb]VMT01 enhances tumor infiltrating lymphocytes in B16-F10 melanoma. (**A**) Lymphocytes were gated to exclude non-lymphocyte populations based on forward and side scatter (FSC and SSC) and stained for live/dead discriminator, CD45, CD19, CD3, CD4, and CD8a; (**B**) FACS analysis of CD45, CD19, CD3, CD4, and CD8a lymphocytes in control for control (*n* = 5) vs. ^212^Pb α-therapy (*n* = 4) treated B16-F10 tumor. Statistical analysis: n.s. non-significant; * *p* < 0.05; ** *p* < 0.01.

To develop a more detailed understanding of the tumor-specific immune response to [^212^Pb]VMT01, changes in tumor-infiltrating lymphocytes (TILs) was measured in [^212^Pb]VMT01 treated B16-F10 tumors. For these experiments, C57BL6 mice bearing B16-F10 tumors were treated with 1.4 MBq [^212^Pb]VMT01. TILs were analyzed 7 days post treatment by flow cytometry using CD45 for leukocytes, CD3 for T cells, CD19 for B cells, CD4 for helper T cells, CD8 for cytotoxic T cells (Figure 6A). Treatment with 1.4 MBq [^212^Pb]VMT01 significantly enhanced the infiltration of CD45+ leukocytes and CD3^+^ T cells compared with control animals (Figure 6B). Among CD45+ leukocytes, CD3^+^ T cells was increased to 39% by [^212^Pb]VMT01 compared with 26% in control animals (Figure 6B). Specifically, within the T cell population, [^212^Pb]VMT01 induced greater tumor infiltrating CD4^+^ helper T cells (63%) and CD8^+^cytotoxic T cells (29%) compared with control animals (Figure 6B). These data demonstrate an immunomodulating effect of [^212^Pb]VMT01 within the melanoma tumor microenvironment. 

## 4. Discussion

In this study, we demonstrated a cooperative anti-tumor effect arising from the combination of ICIs and systemic targeted α-particle radiotherapy using ^212^Pb labeled MC1R-targeted peptide [^212^Pb]VMT01. We have previously reported several cyclic α-MSH analogs that were cyclized via Cu-catalyzed “click” chemistry [49]. With the conjugation of bifunctional chelators, VMT01 were radiolabeled with bivalent radiometals (^203/212^Pb^2+^), allowing for the employment of [^203^Pb]VMT01 as surrogate to determine the injected radioactivity of therapeutic [^212^Pb]VMT01. The injected radioactivity was determined using 11 Gy in kidney dose as a maximum threshold, based on a previous study of α-TRT using a receptor targeted peptide ([^213^Bi]DOTATATE) in which 11 Gy in kidney was identified as the LD5 in athymic nude mice [53]. Of note, the injected radioactivity calculated from 11 Gy in kidney was not the maximal tolerated dose, considering that Miao et al. reported injection of up to 7.4 MBq ^212^Pb radiolabeled peptide in C57BL6 mice without observation of significant toxicities [56]. In this study, acute toxicity was judged by the change in body weight. No significant toxicity was observed in any treatment cohort including the combination of [^212^Pb]VMT01 and ICIs. [^212^Pb]VMT01treatment showed superior efficiency in both tumor-killing and immunogenicity (including 43% complete response rate in combination with ICIs) that relied on intact adaptive immunity. In the genetic modified Rag1KO mice, the immunogenicity of [^212^Pb]VMT01 was completed absent as a result of depleted adaptive immunity. Along with the in vivo evidence, FACS assays focusing on effector T cells demonstrated enhanced TILs, especially CD^8+^ T cells and CD^4+^ T cells in [^212^Pb]VMT01-treated melanoma tumors, indicating strong immunogenic effect of [^212^Pb]VMT01 α-TRT. Meanwhile, Morris et al. demonstrated that the presence of untreated distal tumors jeopardized the synergy between EBRT and immunotherapy in primary irradiated tumors via T_reg_ cell-mediated immunosuppression [30], emphasizing the importance of delivering radiation dose, even partial dose [37,57], to all sites and therefore create as many “hot tumor” sites as possible. Further studies are needed demonstrate the efficacy of combination of [^212^Pb]VMT01 and ICIs in preclinical models with multiple tumor sites that displays heterogeneous expression of MC1R.

The immunogenicity of [^203^Pb]VMT01 could be attributable to its unique high linear energy transfer (LET, keV/micron) and resulted relative biological effectiveness (RBE). The interaction of α-particles in tissues leads to induction of condensed ionization along single relative short mean free path (maximum 100 micron) [58]. As a result, not only do α-particle interactions result in elevated levels of cellular damage (resulting in an increase in tumor associated neoantigens), but also result in a higher probability induced DNA double strand breaks (DSB) at low total absorbed doses [58]. On the other hand, low-LET radiation (i.e., β-particles) require higher absorbed doses to achieve similar levels of DNA DSB. Meanwhile, studies have reported that α-particles induced more apoptotic cell death immediately after irradiation, compared with low LET radiation, which also contributes to enhanced neoantigen presentation [59,60]. Compared with conventional radiotherapy like external beam radiotherapy (i.e., EBRT), the dose-dependent immunogenicity of α-particle radiation is still not fully understood. Recent studies by Vanpouille-Box et al. described a 8–12 Gy dose in tumor as “sweet spot” for the immunogenicity of EBRT (i.e., underdosing was less immunogenic and overdosing was immunosuppressive of cellular machineries) [61] and results presented here suggest that the dosing regimen for α-TRT will be an important parameter for future studies. Therefore, more research will be required to develop a thorough understanding of the dosing regimens of high LET α-particle (i.e., optimal total dose, dose rate, timing of doses) that lead to the most robust tumor-specific immunity and complete responses to treatments, alone and in combination with ICIs.

Further suggesting the importance of dosing regimen, we observed that suboptimal therapeutic outcomes were achieved when ICIs were combined with fractionated administration of [^212^Pb]VMT01. Interestingly, the efficacy of [^212^Pb]VMT01 monotherapy was not affected by fractionation, indicating that the tumor-killing effectiveness of α-TRT does not rely on fractionation as is observed for EBRT [58]. However, only the single injection of [^212^Pb]VMT01 induced potent anti-tumor cooperation with ICIs that led to significant complete tumor regression. Several factors might be considered in these observations. First, studies have demonstrated that ideal tumor response to ICIs is achieved when tumor burden is smallest [62,63]. Thus, it may be that in an aggressive immunotolerant melanoma model, such as the B16-F10 melanoma tumor [64], the initial tumor dose imparted by [^212^Pb]VMT01 must be sufficient to suppress the expansion of tumor size in order to allow for the activation of anti-tumor immunity. In this context, partial doses in each fraction might have led to inadequate control of the fast-growing tumor, which eventually overwhelmed the effectiveness of ICIs. Second, pre-existing TILs are important biomarkers for response to ICIs [65,66]. In this study, we observed significantly enhanced TILs in the B16-F10 tumors seven days post [^212^Pb]VMT01. Chen et al. observed significant influx of TILs in B16 tumors on day 14 post irradiation [67], whereas Morris et al. found infiltrated CD^8+^ T cells in B78 tumor on 12 days post irradiation [30]. Thus, the compromised immunogenicity of fractionated [^212^Pb]VMT01 might be attributable to the suboptimal influx of TILs upon the introduction of ICIs. Furthermore, it was observed that the TILs present in tumor microenvironment were also prone to be depleted by [^212^Pb]VMT01 delivered in later fractions. Third, off-target expression of MC1R expression has been reported in monocytes, macrophages, lymphocytes, and neutrophils [68,69]. Thus, it is also possible that later fractions [^212^Pb]VMT01 delivered B16-F10 tumors imparted radiation dose to these intratumoral immune cells and thereby dampened the immunogenic effect. Whether the expression of MC1R on these immune cells is significant to cause immunosuppression by MC1R-TRT remains unknown and is a subject of further research.

Immunogenic cell death is defined as a specific type of apoptotic cell death that triggers adaptive immune immunity [70]. Typically, immunogenic cell death is associated with expression of surface calreticulin, release of HMGB1, release of ATP, whereas vaccination and tumor re-challenge assays have been considered as a standard in vivo approach to validating immunogenic cell death inducers [70]. In this study, the immunogenicity of [^212^Pb]VMT01 was further determined by vaccination and tumor re-challenge assays, in which melanoma cells were killed by [^212^Pb]VMT01 in cell culture flasks and then injected subcutaneously as cell-based vaccine in C57BL6 mice seven days before re-challenge with naïve melanoma cells. Slower growth of re-challenging tumors was observed in vaccinated animals compared with control cohorts. However, no complete tumor rejection was observed. This might be attributable to insufficient efficacy in one injection of cell-based vaccine, whereas stronger anti-tumor immune reaction might require multiple doses of vaccines (i.e., 2–3 doses) using more [^212^Pb]VMT01 treated cells. Along with the vaccination assays, [^212^Pb]VMT01 was capable of sensitizing immunotolerant melanoma cells to ICI treatments. The exposure to [^212^Pb]VMT01 in vitro led to generation of ICI-sensitive YUMM-PR and B16-PR cells. Generally, radiotherapy has been recognized as a potent inducer of immunogenic cell death that synergizes the efficacy of ICIs. A number of mechanistic pathways are known to be involved in the enhanced anti-tumor immune response that is induced by ionizing radiation. These include induction of the release of DNA and RNA into cytoplasm; induced Type I IFN responses; promotion of the release of danger signals such as damage-associated molecular patterns (DAMPs) [71,72]; activation of the STING signaling pathway [73]; induction of increased expression of major histocompatibility complex class I (MHC I) proteins on the cancer cell surface [71,74]; and enhanced presentation of tumor-associated antigens to immune systems via antigen presenting cells [71,74,75,76]. More importantly, the delivery of radiation doses to multiple tumor sites has been considered beneficial to overcome tumor heterogeneity and immunotolerance by creating more “hot” tumor sites [36,37]. Along these lines, targeted radionuclide therapy (TRT) such as [^212^Pb]VMT01 is emerging as an effective approach to systemically deliver α-particle radiation that not only efficiently eliminates micrometastasis, but also induces anti-tumor immunity to enhance the efficacy of immunotherapies in a cooperative, potentially synergistic manner.

## 5. Conclusions

In this study, ^212^Pb radiolabeled peptide [^212^Pb]VMT01 targeting MC1R was used to deliver α-particle radiation to melanoma cells. Robust anti-tumor cooperation between [^212^Pb]VMT01 and systemic ICIs immunotherapy was observed in preclinical melanoma models. This cooperation relies on the intact adaptive immunity and immunogenicity of [^212^Pb]VMT01. In addition, we have demonstrated that [^212^Pb]VMT01 induces immunogenic cell death, tumor infiltrating lymphocytes, and sensitizes immunotolerant melanoma tumor to ICIs treatments.

## 6. Patents

VMT01 is the subject under US Patent App. 16/312,846.

## Figures and Tables

**Table 1:** Biodistribution of [^203^Pb]VMT01 in B16-F10 melanoma xenograft model.

	0.5 h		1.5 h		3 h		6 h		24 h	
	Average	Std. Dev	Average	Std. Dev	Average	Std. Dev	Average	Std. Dev	Average	Std. Dev
Blood	1.12	0.34	0.08	0.02	0.03	0.01	0.02	0.01	0.01	0.00
Heart	0.66	0.30	0.08	0.03	0.05	0.01	0.03	0.01	0.02	0.00
Liver	0.64	0.25	0.43	0.12	0.31	0.19	0.37	0.17	0.22	0.06
Spleen	0.58	0.14	0.17	0.01	0.12	0.03	0.11	0.04	0.09	0.01
Lungs	2.80	0.66	2.98	2.55	1.34	1.57	1.00	0.34	0.20	0.16
Kidneys	12.78	2.97	6.06	1.72	5.98	1.38	5.32	1.55	2.59	1.39
Tumor	5.47	1.05	8.90	6.30	4.50	1.71	3.76	0.77	1.68	0.46
Muscle	0.73	0.29	0.09	0.04	0.04	0.02	0.03	0.01	0.02	0.00
Skin	2.23	1.49	0.27	0.05	0.15	0.03	0.17	0.06	0.09	0.01
Brain	0.06	0.02	0.02	0.01	0.01	0.00	0.01	0.00	0.01	0.00
Testes	3.23	3.93	0.10	0.04	0.04	0.01	0.04	0.01	0.03	0.01

## Data Availability

Data presented in this study may be available through communication with the corresponding author.

## Supplementary Materials

The following are available online at https://www.mdpi.com/article/10.3390/cancers13153676/s1, Figure S1: No acute toxicity was observed in C57BL6 mice that received combination of [^212^Pb]VMT01 and ICIs; Body weights of animals were monitored twice a week until endpoint was reached. No acute toxicity was observed. Three animals survived until the conclusion of the study on day 80 without significant weight loss.
