# Supplementary material for: Targeted Alpha-Particle Radiotherapy and Immune Checkpoint Inhibitors Induces Cooperative Inhibition on Tumor Growth of Malignant Melanoma
Source: Cancers (Basel). 2021 Jul 22;13(15):3676. doi: 10.3390/cancers13153676 (PMC8345035; doi:10.3390/cancers13153676)
Supplement: Supplementary file 1 [file cancers-13-03676-s001.zip › cancers-1291372-supplementary.pdf]

cancers

MDPI

Targeted Alpha-particle Radiotherapy and Immune Checkpoint Inhibitors Induces Cooperative Inhibition on Tumor Growth of Malignant Melanoma

Mengshi Li, Dijie Liu, Dongyoul Lee, Yinwen Cheng, Nicholas J. Baumhover, Brenna M. Marks, Edwin A. Sagastume, Zuhair K. Ballas, Frances L. Johnson, Zachary S. Morris and Michael K. Schultz

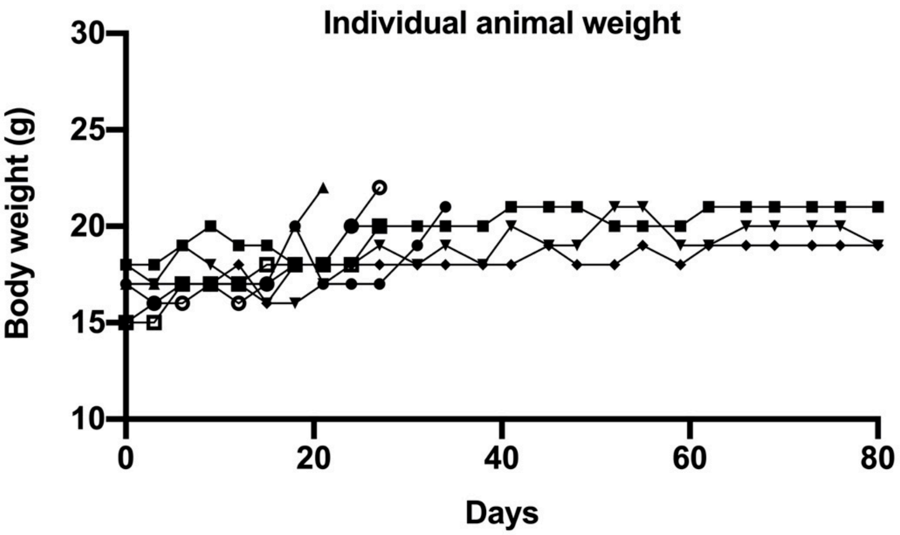

Figure S1. No acute toxicity was observed in C57BL6 mice that received combination of [^{212}Pb]VMT01 and ICIs; Body weights of animals were monitored twice a week until endpoint was reached. No acute toxicity was observed. 3 animals survived until the conclusion of the study on day 80 without significant weight loss.
